# Time-varying maximum capacity path problem with zero waiting times and fuzzy capacities

**DOI:** 10.1186/s40064-016-2654-y

**Published:** 2016-07-04

**Authors:** G. H. Shirdel, H. Rezapour

**Affiliations:** Department of Mathematics, Faculty of Basic Sciences, University of Qom, Qom, Iran

**Keywords:** Time-varying network, Maximum capacity path, Fuzzy numbers

## Abstract

In this paper, the maximum capacity path problem in time-varying network is presented, where waiting times at vertices are not allowable. Moreover, the capacities are considered the generalized trapezoidal fuzzy number. An exact algorithm is proposed which can find a optimal solution of problem subject to the time of path is at most T, where T is a given time horizon.

## Background

The maximum capacity path (MCP) problem is to find a path between two vertices such that the capacity of the path is maximized, where the capacity of a path is defined as the minimum of the capacities of the arcs and vertices on this path. If waiting times at vertices are not allowable, then the capacity of a path is defined as the minimum of the capacities of the arcs. The MCP problem was introduced by Pollack (1960). He applied the cubic shortest path algorithm to solve this problem. The MCP in undirected graphs was surveyed by Hu (1961). He proposed an algorithm in $O(n^{2} )$ time by simply taking the paths in a maximum spanning tree. We encourage the reader to study Lawler (1976), Ichimori et al. (1979), Hansen (1980), Gabow (1985), Berman and Handler (1987), Punuen (1991), Charnsethikul and Virojsailee (2000), Vassilevska et al. (2007) and Martens and Skutella (2009) for algorithms, techniques and properties of MCP in static networks. Arenas et al. (2001) studied network transport capacity, which is known to be measured by the critical value of the transport capacity at the phase transmission from free flow to congestion. Ramasco et al. (2010) surveyed optimization of transport protocols with path constraints in complex network. They proposed a protocol optimization technique that is applicable to both weighted and unweighted graphs. Some efficient routing strategies that can significantly enhance the transport capacity of network were discussed by Kawamoto and Igarashi (2012). Bisla and Singh (2013) studied maximum capacity path problem in mobile ad-hoc network, which include the multi hop transmission of path packets high dynamic topology and limited bandwidth. Gang et al. (2015) considered maximum transport capacity of network. They demonstrated that any network has a maximum transport capacity largely depending on structured properties of the network. The MCP within time constraint was studied by Nopparat (1997). Cai et al. (2007) surveyed MCP in time-varying network, where the problem parameters may change overtime.

In the literature, several algorithms were described to find MCP optimal solutions. They considered MCP problem in static network or time-varying network with real and certain parameters. In the routine life applications, there always exist uncertainty about the parameters of network flows problem. In this paper, a new algorithm is proposed for solving MCP in time-varying network by assuming that waiting times at vertices are zero. Moreover, we consider arc capacities are trapezoidal fuzzy numbers. The following advantages are obtained by using the proposed algorithm for finding the fuzzy optimal solutions:

1. The proposed algorithm is straightforward to realize and apply.
2. The MCP proposed algorithm in fuzzy time-varying is not applied goal and parametric programming techniques.
3. The MCP proposed algorithm does not need to much knowledge of fuzzy logic.
4. The proposed approach can be easily found the optimal solutions, where these solutions are trapezoidal fuzzy numbers.

We will study MCP problem, where the parameters of the network may change over time. Specifically, the transit $b(i,j,t)$ to traverse an arc $(i,j)$ and the capacity $\tilde{u}(i,j,t)$ of the arc $(i,j)$ are functions of the departure time $t$ at the vertex $i$. Moreover, we will consider the transit capacity $\tilde{u}(i,j,t)$ is generalized trapezoidal fuzzy umber and waiting at the vertex $i$ is not allowed. The problem is to determine the maximum capacity path from the source $s$ to the pre-specified vertex, subject to the total travel time of the path is not greater than a given time horizon $T$.

The fuzzy basic definitions, the necessary arithmetic operations of fuzzy numbers and the time- varying network preliminaries are studied in section preliminaries. Then, two theorems are proved for solving the MCP problem and the algorithm is presented, which is worked based on theorems.

## Fuzzy preliminaries

In this section, some fuzzy basic definitions and arithmetic operations and time-varying network flow definitions are briefly presented.

### **Definition 1**

(Kaufmann and Gupta 1988) The characteristic function $\mu_{A} (x)$ of a crisp set $A \subseteq X$ assigns a value either 0 or 1 to each member in $X$. This function can be generalized to a function $\mu_{{\tilde{A}}}$ such that the value assigned to the element of the universal set $X$ fall within a specified range i.e. $\mu_{{\tilde{A}}} (x):\,X \to [0,1]$. The assigned value indicate the membership grade of the element in the set A. The function $\mu_{{\tilde{A}}}$ is called the membership function and the set $\tilde{A} = \left\{ {\left( {x,\mu_{{\tilde{A}}} (x)} \right);x \in X} \right\}$ defined by $\mu_{{\tilde{A}}} (x)$ for each $x \in X$ is called a fuzzy set.

### **Definition 2**

(Kaufmann and Gupta 1988) A fuzzy set $\tilde{A} = \left\{ {\left( {a,b,c,d} \right)\left| {a,b,c,d \in R} \right.} \right\}$ defined on the universal set of real numbers ${\mathbb{R}}$, is said to be a fuzzy number if its membership function has the following characteristics:

1. $$\mu_{{\tilde{A}}} :\,{\mathbb{R}} \to [0,1]$$ is continuous.
2. $$\mu_{{\tilde{A}}} (x) = 0$$ for all $$x \in ( - \infty ,a] \cup [d,\infty )$$.
3. $\mu_{{\tilde{A}}} (x)$ is strictly increasing on $[a,b]$ and strictly decreasing on $[c,d]$.
4. $\mu_{{\tilde{A}}} (x) = 1$ for all $x \in [b,c]$, where $a < b < c < d$.

### **Definition 3**

(Kaufmann and Gupta 1991) A fuzzy number $\tilde{A} = (a,b,c,d)$ is said to be a trapezoidal fuzzy number if its membership function is given by:

1 $$\mu_{{\tilde{A}}} (x)\left\{ {\begin{array}{*{20}l} {\frac{(x - a)}{(b - a)};} \hfill &\quad {a \le x < b} \hfill \\ {1;} \hfill &\quad {b \le x \le c} \hfill \\ {\frac{(x - d)}{(c - d)};} \hfill &\quad {c < x \le d} \hfill \\ {1;} \hfill &\quad {otherwise} \hfill \\ \end{array} } \right.$$

### **Definition 4**

(Chen and Chen 2007) A fuzzy set $\tilde{A} = \left\{ {\left( {a,b,c,d;w} \right)\left| {a,b,c,d \in R} \right.,w \in R^{ + } } \right\}$ defined on the universal set of real numbers ${\mathbb{R}}$, is said to be generalized fuzzy number if its membership function has the following characteristics:

1. $$\mu_{{\tilde{A}}} :\,{\mathbb{R}} \to [0,w]$$ is continuous.
2. $$\mu_{{\tilde{A}}} (x) = 0$$ for all $$x \in ( - \infty ,a] \cup [d,\infty )$$.
3. $\mu_{{\tilde{A}}} (x)$ is strictly increasing on $[a,b]$ and strictly decreasing on $[c,d]$.
4. $\mu_{{\tilde{A}}} (x) = w$ for all $x \in [b,c]$, where $0 < w \le 1$.

### **Definition 5**

(Chen and Chen 2007) A fuzzy number $\tilde{A} = (a,b,c,d;w)$ is said to be a generalized trapezoidal fuzzy number if its membership function is given by:

2 $$\mu_{{\tilde{A}}} (x) = \left\{ {\begin{array}{*{20}l} {w\frac{(x - a)}{(b - a)};} \hfill &\quad {a \le x < b} \hfill \\ {w;} \hfill &\quad {b \le x \le c} \hfill \\ {w\frac{(x - d)}{(c - d)};} \hfill &\quad {c < x \le d} \hfill \\ {0;} \hfill &\quad {otherwise} \hfill \\ \end{array} } \right.$$

### **Definition 6**

(Chen and Chen 2007) Let $\tilde{A}_{1} = (a_{1} ,b_{1} ,c_{1} ,d_{1} ;w_{1} )$ and $\tilde{A}_{2} = (a_{2} ,b_{2} ,c_{2} ,d_{2} ;w_{2} )$ be two generalized trapezoidal fuzzy umbers then arithmetic operations between $\tilde{A}_{1}$ and $\tilde{A}_{2}$ can be defined as follows:

1. $$\tilde{A}_{1} + \tilde{A}_{2} = \left\{ {a_{1} + a_{2} ,b_{1} + b_{2} ,c_{1} + c_{2} ,d_{1} + d_{2} ;\hbox{min} \left( {w_{1} ,w_{2} } \right)} \right\}$$
2. $$\tilde{A}_{1} - \tilde{A}_{2} = \left\{ {a_{1} - d_{2} ,b_{1} - c_{2} ,c_{1} - b_{2} ,d_{1} - a_{2} ;\hbox{min} \left( {w_{1} ,w_{2} } \right)} \right\}$$
3. $$\lambda \tilde{A}_{1} = \left\{ {\begin{array}{*{20}l} {(\lambda a_{1} ,\lambda b_{1} ,\lambda c_{1} ,\lambda d_{1} ;w_{1} );\quad \lambda > 0} \hfill \\ {(\lambda d_{1} ,\lambda c_{1} ,\lambda b_{1} ,\lambda a_{1} ;w_{1} );\quad \lambda < 0} \hfill \\ \end{array} } \right.$$

The ranking function is applied to compare fuzzy numbers. They can be defined as follows:

### **Definition 7**

(Mahapatra and Roy 2006) Let $\tilde{A}_{1} = (a_{1} ,b_{1} ,c_{1} ,d_{1} ;w_{1} )$ and $\tilde{A}_{2} = (a_{2} ,b_{2} ,c_{2} ,d_{2} ;w_{2} )$ be two generalized trapezoidal fuzzy umbers, $\Re :F(R) \to R$ is a ranking function, where $F(R)$ is a set of fuzzy numbers defined on set of real numbers, which maps each fuzzy number into the real line where a natural order exists i.e.,

1. $\tilde{A}_{1} > \tilde{A}_{2}$ if and only if $\Re (\tilde{A}_{1} ) > \Re (\tilde{A}_{2} )$
2. $\tilde{A}_{1} < \tilde{A}_{2}$ if and only if $\Re (\tilde{A}_{1} ) < \Re (\tilde{A}_{2} )$
3. $\tilde{A}_{1} = \tilde{A}_{2}$ if and only if $\Re (\tilde{A}_{1} ) = \Re (\tilde{A}_{2} )$

Moreover, let $w = \hbox{min} (w_{1} ,w_{2} )$ then ranking functions $\Re (\tilde{A}_{1} )$ and $\Re (\tilde{A}_{2} )$ are defined as $\Re (\tilde{A}_{1} ) = w(a_{1} + b_{1} + c_{1} + d_{1} )/4$ and $\Re (\tilde{A}_{2} ) = w(a_{2} + b_{2} + c_{2} + d_{2} )/4$, respectively. Moreover, we let $\tilde{0} = \tilde{A} = (a_{1} ,b_{1} ,c_{1} ,d_{1} )\, \Leftrightarrow \,a_{1} = 0,b_{1} = 0,c_{1} = 0,d_{1} = 0$ and $\tilde{\infty } = \tilde{A}\, \Leftrightarrow \,\Re (\tilde{A}) = \infty$.

### *Remark 1*

(Kaur and Kumar 2012) Let $\tilde{A}_{i} ;\,i = 1,2, \ldots ,n$ be a set of generalized trapezoidal fuzzy numbers. If $\Re (\tilde{A}_{k} ) \le \Re (\tilde{A}_{i} )$ for all $i$, then the generalized trapezoidal fuzzy number $\tilde{A}_{k}$ is the minimum of $\tilde{A}_{i} ;\,i = 1,2, \ldots ,n\,$. Moreover, If $\Re (\tilde{A}_{k} ) \ge \Re (\tilde{A}_{i} )$ for all $i$, then the generalized trapezoidal fuzzy number $\tilde{A}_{k}$ is the maximum of $\tilde{A}_{i} ;\,i = 1,2, \ldots ,n$.

## Time-varying network flow definitions

Consider a directed time-varying network $G(V,A,b,u)$, where $V$ is the set of vertices and $A$ is the set of arcs with $\left| A \right| = m$, $\left| V \right| = n$. The transit time $b(i,j,t)$ and the fuzzy capacity $\tilde{u}(i,j,t)$ are associated with each arc $(i,j) \in A$, respectively such that $t$ is the departure time from vertex $i$ on arc $(i,j)$. Moreover, $b(i,j,t)$ and $\tilde{u}(i,j,t)$ are the functions of discrete time $t = 0,1, \ldots ,T$, where $T$ is a given positive integer. Moreover, consider waiting at any vertex is not allowed.

### **Definition 8**

Suppose a time-varying path from $i_{1}$ to $i_{k}$ is specified by $P\left( {i_{1} - i_{2} - \cdots - i_{k} } \right)$ with zero waiting times at vertices. Consider $\alpha (i_{l} )$ be an arrival time into the vertex $i_{l}$ on $P\left( {i_{1} - i_{2} - \cdots - i_{k} } \right)$ such that $\alpha (i_{1} ) = t_{1} \ge 0$ and:

3 $$\alpha (i_{l} ) = \alpha (i_{l - 1} ) + b(i_{l - 1} ,i_{l} ,\tau (i_{l - 1} ))\quad for\;\; 2 \le l \le k$$

where $\tau \left( {i_{l - 1} } \right)$ is departure time from vertex $i_{l - 1}$ for $2 \le l \le k$ on $P\left( {i_{1} - i_{2} - \cdots - i_{k} } \right)$ and we have:

4 $$\tau (i_{l - 1} ) = \alpha (i_{l - 1} ) \quad for\;\;2 \le l \le k$$

Meantime, we let $\alpha (s) = 0$ for source vertex $s$.

### **Definition 9**

Let $P\left( {i_{1} - i_{2} - \cdots - i_{k} } \right)$ be a time-varying path from $i_{1}$ to $i_{k}$, where waiting at any vertex is not allowed, then:

1. The time of path $P\left( {i_{1} - i_{2} - \cdots - i_{k} } \right)$ is determined by $\alpha (i_{k} ) - \alpha (i_{1} )$. So, the time of path $P\left( {i_{1} = s - i_{2} - \cdots - i_{k} } \right)$ is $\alpha (i_{k} )$.
2. The path $P\left( {i_{1} - i_{2} - \cdots - i_{k} } \right)$ has time at most $t$ if $\alpha (i_{k} ) - \alpha (i_{1} ) \le t$ and has time exactly $t$ if $\alpha (i_{k} ) - \alpha (i_{1} ) = t$.

### **Definition 10**

The capacity of a time-varying path with zero waiting times is defined as the minimum of the capacities of the arcs on this path. Let $\xi (i,t)$ be the maximum capacity of the path from source vertex $s$ to vertex $i$ of time exactly $t$ subject to the waiting time at any vertex $j$ on the path is not allowable. If this path does not exist, let $\xi (i,t) = 0$.

### **Definition 11**

Define $P\left( {i_{1} - i_{2} - \cdots - i_{k} } \right)$ as a time-varying path with maximum capacity from $i_{1}$ to $i_{k}$ within time exactly $t$, if for each time-varying path $P^{{\prime }}$ from $i_{1}$ to $i_{k}$ within time $t$ and capacity $\xi (P^{{\prime }} )$, we have: $\xi (P) \ge \xi (P^{{\prime }} )$.

## Main results

### Time-varying MCP problem with fuzzy capacities

The MCP problem is to find a path between two vertices such that the capacity of the path is maximized, where the capacity of a path is defined as the minimum of the capacities of the arcs on this path. In this section, the time-varying MCP problem is studied, where the problem parameters may change over time, where the capacity $\tilde{u}(i,j,t)$ of the arc $(i,j)$ is generalized trapezoidal fuzzy number and is function of the departure time $t$ at the vertex $i$. Moreover, a transit time $b(i,j,t)$ to traverse an arc $(i,j)$ is considered positive real functions of the departure time $t$ at the vertex $i$. Waiting at the vertex $i$ is not allowed. The problem is to determine the maximum capacity path from the source $s$ to the pre-specified vertex, such that the total travel time of the path is not greater than a given time horizon $T$.

#### **Theorem 1**

$\xi (s,0) = \tilde{\infty }$*and*$\xi (j,0) = \tilde{0}$*for*$j \ne s$*. For*$t > 0$*, we have:*

$$\xi (j,t) = \mathop {Max}\limits_{(i,j) \in A} \,\mathop {Max}\limits_{r + b(i,j,r) = t} \,\left\{ {Min\left\{ {\xi (i,r),\tilde{u}(i,j,r)} \right\}} \right\}$$

#### *Proof*

It is clear that $\xi (s,0) = \tilde{\infty }$ and $\xi (j,0) = \tilde{0}$ for $j \ne s$, since all transit times are positive. Now, the theorem is proved by induction on $t$. Consider $t = 1$, therefore the paths of time exactly one can be existed from source vertex $s$ to neighbors of $s$. Moreover, consider $(s,j) \in A$ and $b(s,j,0) = 1$. In this case, the formula holds with $Min\left\{ {\xi (i,r),\tilde{u}(i,j,r)} \right\}$, where $r = 0$ and $i = s$. Assume that the theorem is correct for all $t^{{\prime }} < t$. Consider a vertex $j \ne s$. If $\xi (j,t) = \tilde{0}$, there is nothing to prove. So assume $\xi (j,t) > \tilde{0}$. First, it is shown that there exists a path from $s$ to $j$ of time exactly $t$ with fuzzy capacity $\xi (j,t)$. By the formula, $\xi (j,t) = Min\left\{ {\xi (i,r),\tilde{u}(i,j,r)} \right\}$, for some $i$ such that $(i,j) \in A$ and some $r$ such that $r + b(i,j,r) = t$. By induction, since $r + b(i,j,r) = t$ then $r < t$ and we know that there is a feasible path $P^{{\prime }} (s,i)$ from $s$ to $i$ of time exactly $r$ and capacity $\xi (i,r)$. This path can be extended to vertex $j$ and obtained a path $P$ such that the time of $P$ is exactly $t$. The fuzzy capacity of $P$ is $Min\left\{ {\xi (i,r),\tilde{u}(i,j,r)} \right\} = \xi (j,t)$. This proves the claim. We now prove that $\xi (j,t)$ is the maximum fuzzy capacity path from $s$ to $j$ of time exactly $t$. Let $P(s = i_{1} ,i_{2} , \ldots ,i_{k} = j)$ be a maximum capacity path from $s$ to $j$ of time exactly $t$. Therefore, $\xi (P) \ge \xi (j,t)$. Let $i$ be the predecessor node of $j$ on this path. Let $r$ be the time of the subpath $P^{\prime}$ from $s$ to $i$ and let $\xi (P^{\prime})$ be the capacity of $P^{\prime}$. By definition, $r + b(i,j,r) = t$, implying that $r < t$ since $b(i,j,r) > 0$. Thus, by induction, we have: $\xi (P^{\prime}) \le \xi (i,r)$. By definition, $\xi (P) = min\left\{ {\xi (P^{\prime}),l(i,j,t)} \right\} \le min\left\{ {\xi (i,u),l(i,j,t)} \right\} \le \xi (j,t)$. Therefore $\xi (P) = \xi (j,t)$, since $P$ is a maximum fuzzy capacity path of time exactly $t$.□

#### **Theorem 2**

*Define*$\xi^{*} (i)$*as the fuzzy capacity of a maximum time*-*varying path from vertex*$s$*to the vertex*$i$*of time at most*$T$*, where waiting at vertices are not allowed, then:*

$$\xi^{*} (i) = \mathop {Max}\limits_{0 \le t \le T} \,\xi (i,t)$$

#### *Proof*

By definitions of $\xi^{*} (i)$, the purpose is to find maximum $\xi (i,t)$ on all of time steps t, such that $0 \le t \le T$, therefore we have: $\xi^{*} (i) = \mathop {Max}\limits_{0 \le t \le T} \,\xi (i,t)$.□

The following algorithm can find the optimal solution of problem. In the first, the values of $r + b(i,j,r)$ for all $r = 1,2, \ldots ,T$ and all arcs $(i,j) \in A$, are sorted by algorithm. Then, the recursive relation as given in theorem 1 is applied to compute $\xi (j,t)$ for all $j \in V$ and $t = 1,2, \ldots ,T$. The steps of the algorithm are described as below:
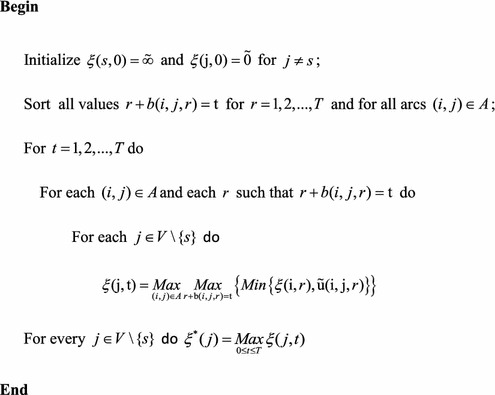


## Results

### *Example 1*

**Fig. 1:**
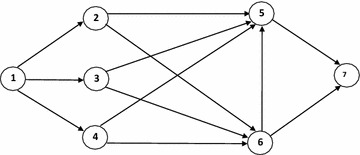
Time-varying network *G*

Consider a time-varying network $G$ as shown in Fig. 1, where waiting times are assumed zero at the vertices. The problem is to find a maximum path connecting source node 1 and the sink node 7, such that the time of this path is at most $T = 6$.

Let $A = \{ (1,2),\,(1,4),\,(5,7),\,(6,7)\}$ and consider:

$$\hbox{for}\,(i,j) \in A,\quad t = 0,1, \ldots ,6;\quad \left( {\tilde{u}(i,j,t),b(i,j,t)} \right) = \left( {(2,4,5,7;0.8),\,2} \right)$$

Another required information for time-varying network *G* is given in Table 1 as follows:

**Table 1:** Transit times and fuzzy capacities for network G

*t*	$\tilde{u}$, b							
	$\tilde{u}(1,3,t)$	$b(1,3,t)$	$\tilde{u}(2,5,t)$	$b(2,5,t)$	$\tilde{u}(2,6,t)$	$b(2,6,t)$	$\tilde{u}(3,5,t)$	$\,\,b(3,5,t)$
0	(1, 2, 3, 4; 0.5)	1	(2, 3, 4, 5; 0.4)	1	(2, 3, 4, 6; 0.3)	1	(2, 3, 5, 6; 0.6)	3
1	(2, 3, 4, 5; 0.6)	1	(2, 4, 6, 8; 0.3)	2	(1, 2, 3, 4; 0.4)	1	(1, 3, 5, 6; 0.5)	2
2	(1, 3, 5, 7; 0.5)	2	(1, 3, 4, 5; 0.3)	2	(2, 3, 5, 7; 0.4)	2	(2, 4, 5, 7; 0.7)	1
3	(2, 4, 6, 8; 0.4)	2	(2, 3, 4, 6; 0.5)	1	(1, 3, 4, 6; 0.4)	2	(2, 4, 6, 8; 0.6)	2
4	(1, 2, 3, 4; 0.5)	3	(1, 4, 5, 7; 0.6)	3	(2, 3, 5, 6; 0.3)	2	(3, 4, 5, 7; 0.6)	2
5	(1, 2, 3, 5; 0.6)	2	(2, 5, 6, 8; 0.5)	4	(3, 4, 5, 6; 0.3)	3	(2, 3, 4, 7; 0.5)	2
6	(3, 4, 5, 7; 0.5)	3	(1, 3, 5, 7; 0.4)	3	(2, 3, 5, 7; 0.5)	3	(1, 2, 3, 4; 0.6)	3

*t*	$\tilde{u}$, b							
	$\tilde{u}(3,6,t)$	$b(3,6,t)$	$\tilde{u}(4,5,t)$	$b(4,5,t)$	$\tilde{u}(4,6,t)$	$b(4,6,t)$	$\tilde{u}(6,5,t)$	$\,\,b(6,5,t)$
0	(2, 3, 6, 7; 0.4)	3	(3, 4, 5, 6; 0.4)	4	(1, 2, 3, 4; 0.4)	3	(1, 3, 4, 6; 0.6)	4
1	(2, 4, 5, 6; 0.5)	3	(3, 5, 6, 7; 0.4)	3	(2, 3, 4, 6; 0.4)	3	(2, 3, 4, 5; 0.6)	4
2	(2, 4, 6, 8; 0.6)	4	(2, 3, 6, 8; 0.6)	2	(2, 4, 5, 7; 0.3)	2	(1, 4, 5, 7; 0.6)	3
3	(2, 3, 4, 5; 0.6)	2	(2, 4, 6, 7; 0.6)	2	(3, 4, 5, 7; 0.6)	2	(2, 4, 5, 7; 0.7)	2
4	(1, 2, 3, 4; 0.6)	2	(3, 5, 6, 8; 0.7)	2	(3, 5, 6, 7; 0.6)	2	(1, 3, 5, 6; 0.7)	2
5	(2, 4, 6, 8; 0.4)	2	(3, 5, 7, 9; 0.7)	1	(2, 4, 6, 8; 0.4)	2	(3, 4, 6, 7; 0.6)	2
6	(1, 2, 3, 4; 0.6)	1	(1, 3, 4, 7; 0.6)	3	(3, 4, 7, 8; 0.5)	4	(2, 4, 6, 8; 0.6)	2

Applying described algorithm, one may obtain this results: the path $P = (1 - 4 - 5 - 7)$ has maximum fuzzy capacity with $\xi (P) = (2,4,5,7,0.6)$ and time 6. Moreover, three paths $P = (1 - 2 - 6 - 5 - 7)$, $P = (1 - 3 - 6 - 5 - 7)$ and $P = (1 - 4 - 6 - 5 - 7)$ are not feasible, because their time are 8 and are more than $T = 6$.

### *Example 2*

A time-varying network $G\left( {V,A,b,u} \right)$ is called a layered network with $k$ layers if the vertices set $V$ can be partitioned into $k$ subsets $l_{1} , \ldots ,l_{k}$ such that the following conditions hold:

1. For every $i \in l_{s} ,\,s = 1,2, \ldots ,k - 1$: For every arc $(i,j) \in A$ it holds that $j \in l_{s + 1}$.
2. For every $i \in l_{s} ,\,s = 2,3, \ldots ,k$: For every arc $(j,i) \in A$ it holds that $j \in l_{s - 1}$.
3. There are no incoming arcs for vertices in $l_{1}$ and there are no outgoing arcs for vertices $l_{s}$

**Fig. 2:**
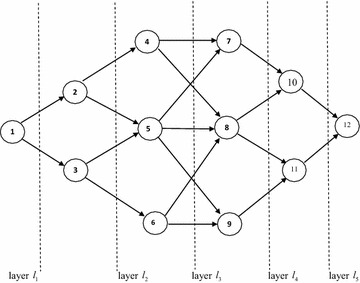
A layered time-varying network *G* for example 2

In Fig. 2, a layered time-varying network is given, where waiting times are considered zero at vertices. Moreover, let $T = 15$ and:

$b(i,j,t) = s$ for $t = 0,1, \ldots ,15$ and for $(i,j) \in l_{s}$ such that $s = 1,2, \ldots ,5$. Information about fuzzy capacities is given as follows:

Let: $$\begin{array}{*{20}l} {A = \left\{ {\left( {1,2} \right)\left( {2,5} \right)\left( {4,7} \right)\left( {10,12} \right)\left( {11,12} \right)} \right\}} \hfill & {B = \left\{ {\left( {2,4} \right)\left( {3,6} \right)\left( {4,8} \right)\left( {5,7} \right)\left( {6,8} \right)} \right\}} \hfill \\ {C = \left\{ {\left( {5,8} \right)\left( {5,9} \right)\left( {6,9} \right)\left( {8,11} \right)} \right\}} \hfill & {D = \left\{ {\left( {1,3} \right)\left( {3,5} \right)\left( {7,10} \right)\left( {8,10} \right)\left( {9,11} \right)} \right\}} \hfill \\ \end{array}$$

For each $(i,j) \in A$ and $0 \le t \le 15$ let $\tilde{u}(i,j,t): = (5,6,7,8;0.5)$.

For each $(i,j) \in B$ and $0 \le t \le 15$ let $\tilde{u}(i,j,t): = (2,3,4,5;0.2)$.

For each $(i,j) \in C$ and $0 \le t \le 15$ let $\tilde{u}(i,j,t): = (6,7,8,9;0.6)$.

For each $(i,j) \in D$ and $0 \le t \le 15$ let $\tilde{u}(i,j,t): = (1,2,3,4;0.7)$.

The following table shows the optimal solutions for Example 2.

By applying mentioned algorithm, the MCP *P* = (1–2–5–8–11–12) from vertex 1 to vertex 12 is obtained by $\xi (P) = \left( {5,6,7,8;0.5} \right)$ and time $t = 15$. The maximum capacity paths for other vertices were shown in Table 2.

**Table 2:** Calculation of optimal solution for example 1

Vertex	$P,T,\xi$			Vertex	$P,T,\xi$		
	Path	Time	$\xi (P)$		Path	Time	$\xi (P)$
1	P(1)	0	$\infty$	7	P(1–2–5–7)	6	(2, 3, 4, 5; 0.2)
2	P(1–2)	1	(5, 6, 7, 8; 0.5)	8	P(1–2)	1	(5, 6, 7, 8; 0.5)
3	P(1–3)	1	(1, 2, 3, 4; 0.2)	9	P(1–2–5–9)	6	(5, 6, 7, 8; 0.5)
4	P(1–2–4)	3	(2, 3, 4, 5; 0.2)	10	P(1–2–5–8–10)	10	(1, 2, 3, 4; 0.7)
5	P(1–2–5)	3	(5, 6, 7, 8; 0.5)	11	P(1–2–5–8–11)	1	(5, 6, 7, 8; 0.5)
6	P(1–3–6)	3	(1, 2, 3, 4; 0.2)	12	P(1–2–5–8–11–12)	15	(5, 6, 7, 8; 0.5)

## Conclusion

In this paper, we concentrated on time-varying maximum capacity path with zero waiting times. We considered the capacity of arcs are fuzzy numbers. The aim of the problem was to find an optimal path from source vertex to target vertex so that the capacity of this path is maximized subject to the time of path is at most T, where T is a given integer. We proved two theorems, presented an algorithm for solving the problem and given two numerical examples.
